# CD36: The Bridge between Lipids and Tumors

**DOI:** 10.3390/molecules29020531

**Published:** 2024-01-21

**Authors:** Xuan Zhou, Manman Su, Jungu Lu, Deming Li, Xinhui Niu, Yi Wang

**Affiliations:** Department of Regenerative Medicine, School of Pharmaceutical Sciences, Jilin University, Changchun 130012, China; zhoux21@mails.jlu.edu.cn (X.Z.); lujg22@mails.jlu.edu.cn (J.L.); lidm23@mails.jlu.edu.cn (D.L.); niuxh22@mails.jlu.edu.cn (X.N.)

**Keywords:** CD36, fatty acid, immunosuppress, lipid, metastasis-initiating cells

## Abstract

It has been found that the development of some cancers can be attributed to obesity, which is associated with the excessive intake of lipids. Cancer cells undergo metabolic reprogramming, shifting from utilizing glucose to fatty acids (FAs) for energy. CD36, a lipid transporter, is highly expressed in certain kinds of cancer cells. High expressions of CD36 in tumor cells triggers FA uptake and lipid accumulation, promoting rapid tumor growth and initiating metastasis. Meanwhile, immune cells in the tumor microenvironment overexpress CD36 and undergo metabolic reprogramming. CD36-mediated FA uptake leads to lipid accumulation and has immunosuppressive effects. This paper reviews the types of FAs associated with cancer, high expressions of CD36 that promote cancer development and progression, effects of CD36 on different immune cells in the tumor microenvironment, and the current status of CD36 as a therapeutic target for the treatment of tumors with high CD36 expression.

## 1. Introduction

Lipids are among the most important nutrients required by the human body. They consist of many different types of molecules, including phospholipids, fatty acids (FAs), triglycerides, sphingolipids, cholesterol, and cholesteryl esters [1]. While the majority of human FAs are obtained from food, the body can also convert sugars and proteins into FAs, a process that takes place primarily in the liver [2]. Moreover, FAs are the primary energy source of cells. During periods of low energy demand, the adipose tissue can store excess FAs as triacylglycerols (TAG) in intracellular lipid droplets (LDs). Such reserves are used as buffers to maintain cellular lipid composition homeostasis or as a source of energy to drive cellular processes [3]. Therefore, certain amounts of lipids must be consumed daily. However, in some cases, excessive intake may lead to the development of a variety of diseases.

Obesity is a severe public health problem and is typically associated with diet. Obesity increases the risk of metabolic diseases and can also lead to premature deaths [4]. It is found that about 25% of adults in industrialized countries are clinically diagnosed as obese and 60% as overweight, and this situation is likely to worsen in the future. Moreover, excess body fat can increase the risk of developing different kinds of cancer, such as esophageal, colorectal, gallbladder, pancreatic, endometrial, and breast cancers, with respect to postmenopausal women, and advanced prostate cancer, with respect to men. Evidence has shown that 4–38% of these cancers, depending on the location of the tumor and the patients’ sex, can be attributed to overweight/obesity [5]. Excessive food intake, especially a high-fat diet, is the most critical factor in the development of obesity [6]. Meanwhile, a high-fat diet can cause many types of cancer, such as ovarian cancer [7], prostate [8], skin [9], breast [10], pancreatic [11], and liver cancers [12].

The Western dietary pattern is considered a typically high-fat diet, characterized by a high intake of refined starches, sugar, red meat, processed red meat, saturated and trans fats, and a low intake of fruits, vegetables, and whole grains [13]. According to the International Agency for Research on Cancer and the World Cancer Research Fund/American Institute for Cancer Research, red meat consumption can increase the risk of lung, pancreatic, and prostate cancers [14]. In 2021, Huang et al. published a comprehensive review, using 72 meta-analyses in order to demonstrate that red meat consumption is associated with a high risk of overall cancer mortality and non-Hodgkin’s lymphoma (NHL). Additionally, red meat consumption can also lead to the development of multiple cancers, including bladder, breast, colorectal, endometrial, esophageal, gastric, lung, and nasopharyngeal cancers [14]. The consumption of processed meats may increase the risk of overall cancer mortality and render people more susceptible to cancers, including NHL, bladder, breast, colorectal, esophageal, gastric, nasopharyngeal, oral and oropharyngeal cancers, and prostate cancer [15]. A prospective study found that the consumption of 100% fruit juices and sugar-sweetened beverages was positively associated with overall cancer risk, and the consumption of sugar-sweetened drinks specifically was positively associated with breast cancer risk [16]. Other types of foods, such as fried foods and refined grains, are also related to the risk of cancer, as shown in Table 1.

Increasing evidence has shown that a high-fat diet correlates with cancer occurrence, development, and prognosis. Dysregulated FA metabolism can lead to the development of metabolic disorders and carcinogenesis [42]. Throughout cancer progression, FAs flow into cancer cells via lipid transporters, exemplified by CD36, which is ubiquitously expressed in various cell types, including platelets, mononuclear phagocytes, adipocytes, hepatocytes, myocytes, and certain kinds of epithelial cells [43]. Moreover, there is growing evidence that high expressions of CD36 in certain cancer cells promotes cancer progression and metastasis. A recent study identified a specific cell type, CD44 bright, in human oral cancer samples with high levels of FA receptor CD36 expression. These cells show significantly high metastatic potential when transplanted into mice on a high-fat diet [44]. Therefore, the role of lipids, especially FAs, in cancer development, and the effect of lipid transport on cancer progression, need to be studied.

## 2. Fatty Acids in the Diet and Their Relationship to Cancer

Tumor cells can acquire lipids by both endogenous synthesis and external uptake. The following are fatty acids that can be consumed in diets and their relationship to cancer development.

### 2.1. Saturated Fatty Acids (SFAs)

Saturated fatty acids are a class of fatty acids that do not have an unsaturated bond in the carbon chain and are one of the essential components of lipids. SFAs come mainly from red meats and dairy products [45]. The current consensus among nutritionists is that saturated fats can be consumed in moderation with a balanced diet. There are even some controversial results about SFAs and the risk of cancer. However, most reports point to a diet heavy in saturated fat as a potential tumor promoter. Findings from a meta-analysis indicate that consuming more SFAs in the diet increased the risk of liver cancer [12]. Another study revealed that SFAs from red meats and processed meats were positively related to oral cancer risk [45]. A high intake of SFAs from red meats was associated with an increased risk of lung cancer [46]. Another meta-analysis found that a higher dietary intake of SFAs was associated with a higher risk of breast cancer after menopause [47]. SFAs and cholesterol synergistically promoted prostate cancer stem cell proliferation and contributed to prostate cancer progression [8]. Palmitic acid (PA) is the most common SFA in the body, and people can obtain PA through their diets. Pancreatic cancer risk was strongly associated with the intake of PA [48]. In addition, studies have suggested that such diets may reduce the effectiveness of anticancer treatments [49]. PA enters gastric cancer (GC) cells through its receptor CD36, promoting GC metastasis [50]. A palmitic acid-rich diet fed to mice advanced colon cancer tumor growth [51]. Therefore, an excessive intake of SFAs can increase the risk of cancer and poor prognosis.

### 2.2. Unsaturated Fatty Acids

Unsaturated fatty acids can be categorized according to the number of double bonds in monounsaturated fatty acids (MUFAs) and polyunsaturated fatty acids (PUFAs), where monounsaturated fatty acids contain only one double bond in the carbon chain. In contrast, polyunsaturated fatty acids have two or more double bonds. These two types of unsaturated fatty acids act in diametrically opposed ways.

MUFAs: Among MUFAs, oleic acid (OA) is the human diet’s most abundant representative fatty acid. OA induced Hela cell growth and metastasis by promoting high CD36 expression [52]. It could also enhance the proliferation of breast cancer (MCF7) cells [53]. Moreover, it enabled the invasiveness of gastric cancer cells [54]. OA promoted the invasiveness of the prostate cancer cell line PC3 [55]. However, with a 78% oleic acid content, olive oil contains many bioactive substances, such as polyphenols, with antitumor and anti-inflammatory properties, and its consumption is considered an essential factor in maintaining a healthy lifestyle [56]. Olive oil is the primary source of unsaturated fatty acids in the Mediterranean diet. Many nuts contain mainly MUFAs (mainly OA), and nuts, as an alternative source of MUFAs, may reduce cancer risk [49]. These conclusions encourage people to consume fat from plants.

PUFAs: ω-6 PUFAs and ω-3 PUFAs 22 are two main types of fatty acids [49]. Linoleic acid (LA), an ω-6 polyunsaturated fatty acid, can most often be found in dietary fats and most oils, except flaxseed oil, which contains the highest amount of Alpha-linolenic acid (ALA), the most common ω-3 polyunsaturated fatty acid. PUFAs can also be found in soybean oil, canola oil, and fish [49]. As essential FAs, LA and ALA can lead to several balance-maintaining products in our bodies, such as arachidonic acid (AA, ω-6), eicosapentaenoic acid (EPA, ω-3), and docosahexaenoic acid (DHA, ω-3) [57]. People come to obtain DHA and EPA mainly through their diet (mainly fish oil) [58]. Feeding mice a diet high in omega-6 fatty acids (containing 23% corn oil) promotes pancreatic tumorigenesis in vivo [59]. CT26 mouse colon cancer cells were inoculated on the backs of BALB/c mice, and LA was administered by gavage. The results showed that LA promoted tumor cell growth and induced cancer cell stemness, which in turn promoted tumor metastasis [60]. Nude mice transplanted with human gastric cancer cells (OCUM-2MD3) and then fed LA showed that LA could stimulate gastric cancer cell invasion and peritoneal metastasis by cyclooxygenase (COX)-catalyzed metabolism and ERK activation [61]. In vitro, LA induced the migration and invasion of breast cancer cells via the FFAR4, EGFR, and PI3K-/Akt pathways [62]. Roberto Espinosa-Neira et al. studied LA in MCF10A human mammary epithelial cells and, for the first time in 2011, substantiated that LA could promote an epithelial–mesenchymal transition (EMT)-like process in those cells through activating FAK and NFκB, affecting MMP-2 secretions, inducing cell migration and cancer invasion, and regulating the expression levels of many proteins, such as decreasing E-cadherin expression while increasing Snail1, Snail2, Twist1, Twist2, and Sip1 expressions [63]. There was a positive association among US adults between hepatocellular carcinogenesis (HCC) and the total intake of PUFAs and ω-6 PUFAs. In contrast, the intake of long-chain omega-3 fatty acids and MUFAs was negatively associated with liver cancer [64]. ω-6 PUFAs could enhance the metastatic ability of gastric cancer cells through the COX-2/PGE2 signaling pathway, whereas ω-3 PUFAs serve to inhibit that ability through the COX-1/PGE3 signaling axis [65]. In breast cancer cells, ω-3 PUFAs induced the downregulation of EZH2, thus exerting anticancer effects [66]. In an induced hamster oral cancer model, it was found that low tumor numbers and volumes were associated with low intakes of ω-6/ω-3 fatty acid. Researchers also found that such a decrease in tumor burden in the right buccal pouch tissue also showed a connection with lower levels of NFκB, proliferating cell nuclear antigen and cyclin D1 [67]. A recent analysis suggested that increasing the dietary intake ratio of ω-3/ω-6 PUFAs could benefit breast cancer prevention [68]. Adjusting the balance of dietary ω-3/ω-6 PUFAs might help prevent some cancers.

### 2.3. Trans Fatty Acids (TFAs)

TFAs are MUFAs or PUFAs with one or more double bonds in the trans configuration. Most TFAs derive from hydrogenated fats; only a small part comes from animal oils. With the development of the food industry, TFAs have been widely found in diets such as margarine, cookies, and repeated cooking oils. A high intake of TFAs puts prostate and colorectal cancer (CRC) at higher risk [69]. Industrially produced TFAs (iTFAs) are the primary source of TFAs in the human diet. Early studies on the health risks of TFAs in humans focused on the cardiovascular aspects related to iTFAs [70]. Elaidic acid (EA) is the most representative iTFA. The results of a recent analysis suggest that higher iTFA intake, especially EA, is associated with an increased risk of breast cancer [71]. A piece of evidence indicated that a higher intake of industrial trans-EA is associated with a higher risk of ovarian cancer [72]. An in vivo study pointed out that EA could promote stemness markers in CT26 cells, a mouse CRC cell, thereby increasing their metastasis ability [73]. A study related to NHL found a positive association between TFA intake and the risk of diffuse large B-cell lymphoma (an aggressive NHL subtype) [74]. Another study using hepatitis C virus (HCV) core gene transgenic mice added that a high TFA intake could also lead to tumors in livers, and that multiple signaling pathways were involved, such as NF-κB and NRF2-p62/SQSTM1 signaling, ERK and Wnt/β-catenin pathways [75]. The WHO recommends that the total trans fatty intake should be limited to less than 1% of the total energy intake, which translates to less than 2.2 g/day, based on a 2000-calorie diet.

**Figure 1:**
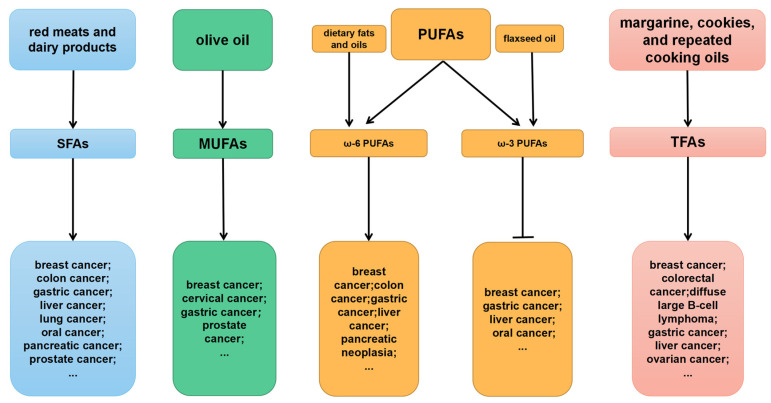
Overview of the relationship among SFAs, MUFAs, PUFAs, and TFAs in cancer. With the exception of ω-3 PUFAs, which can inhibit tumors, most fatty acids have tumor-promoting effects.

An overview of the relationships among SFAs, MUFAs, PUFAs, and TFAs with cancer is shown in Figure 1. Above all, the intake of fatty acids has an essential impact on tumor occurrence, invasion, and metastasis. Therefore, a moderate intake of fatty acids is of great significance for preventing and improving cancer prognosis.

## 3. Metabolic Reprogramming of Tumor Cells

Malignant tumors pose a severe threat to human health. Tumor cells reprogram their metabolic phenotype to meet increased bioenergetic demands and support their malignant behaviors in a nutrient-poor environment, causing their metabolic pattern to be different from that of normal cells. Normal cells derive energy through two main processes: oxidative phosphorylation and glycolysis. Oxidative phosphorylation occurs in the mitochondria and relies on the presence of adequate oxygen. It efficiently converts glucose into ATP, the cellular energy currency. In contrast, glycolysis takes place in the cytoplasm and operates when oxygen availability is insufficient. It breaks down glucose into pyruvate, producing a smaller amount of ATP. These two mechanisms allow normal cells to adapt to varying oxygen levels and generate energy efficiently. The “Warburg effect” defines the glucose metabolism of tumor cells, wherein, regardless of oxygen levels, tumor cells utilize glycolysis to convert pyruvate from glucose into lactate, thereby generating ATP. Hence, tumor metabolism is often referred to as “aerobic glycolysis”. More importantly, cancer cells deplete nutrients and produce immunosuppressive metabolites that interfere with immune cell function [76]. Along with aerobic glycolysis, tumor cells use glutamine, serine, arginine, fatty acids, and lipids to promote their proliferation. Among them, fatty acids are one of the most vital energy sources [77]. The proliferation of cancer cells necessitates the accumulation of substantial lipid quantities, which can be acquired from external sources, or synthesized internally through the adipogenic pathway. Clinical investigations are presently underway to explore the potential of fatty acid synthase inhibitor drugs targeting the fatty acid synthesis pathway. Among these inhibitors, TVB-2640, a small molecule compound, has exhibited inhibitory effects on fatty acid synthase in clinical studies [3]. The reprogramming of fatty acid metabolism is a feature of malignant tumors. Fatty acid uptake, storage, and lipogenesis are upregulated in various cancers, and tumor cells obtain energy through FA oxidation (FAO), which helps promote rapid tumor growth. Specific metabolic activities can participate directly in the transformation processes or support the biological processes that enable tumor growth [78].

**Figure 2:**
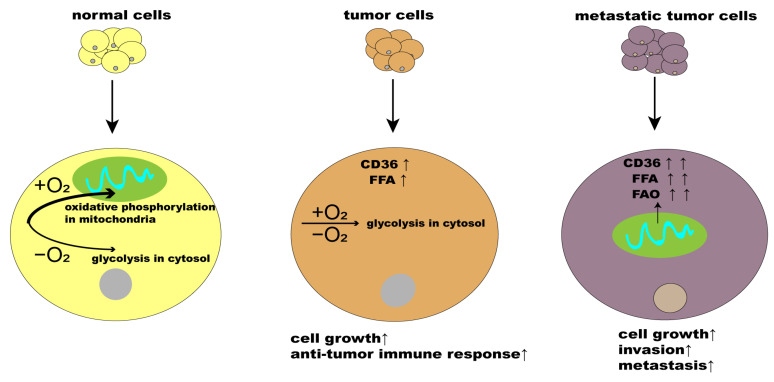
Metabolic characteristics of normal cells, tumor cells, and metastatic tumor cells. Normal cells obtain energy by the oxidative phosphorylation of glucose in the mitochondria cloth in the presence of sufficient oxygen, and glycolysis in the cytoplasm in the presence of insufficient oxygen. In addition to using glucose in glycolysis for energy, tumor cells take up free fatty acids via CD36 and utilize fatty acid functions to meet the demands of high proliferation. Metastatic tumor cells demonstrate an augmented metabolic activity, particularly in glucose and fatty acid metabolism, when compared to primary tumors. This metabolic adaptation enables metastatic tumor cells to enhance fatty acid uptake and metabolism, thereby utilizing fatty acids as an additional energy source to satisfy their elevated energy requirements.

At different stages of cancer development, lipid metabolism shows a generalized enhancement that provides a specific energy source for tumor cells, triggers particular signaling pathways and epigenetic events, and remodels membrane components that favor metastasis [79]. Metastatic tumor cells possess the ability to undergo EMT, which leads to their disparities from normal tumor cells. In this process, metastatic tumor cells obtain skills related to invasion, metastasis, and the resistance to radiotherapy and chemotherapy [80]. During tumor progression and metastasis, fatty acids provide sufficient energy to tumor cells [81]. One study found that sentinel lymph node metastases showed a greater degree of fatty acid accumulation when compared to the primary tumor [82]. Tumor cells optimize their requirements for aggressive progression by switching lipid anabolism and catabolism. Fatty acids are a component of most lipids and have been shown to drive tumor progression [83]. The uptake of exogenous fatty acids into cancer cells could facilitate metastasis [84]. Fatty acid uptake is critical in fatty acid transportation during cell membrane biosynthesis, energy storage, and signaling pathway activation [85]. A transport system that assisted fatty acid translocation involved CD36 and FABPs, working as transporters that collected fatty acids from the surrounding environment and then moved them across the plasma membrane, thus pointing out their crucial position in reorganizing the metabolic phenotype of tumor cells. Recent studies have shown that CD36 expression promoted the metastasis of human oral carcinoma cells, ovarian cancer cells, gastric cancer cells, pancreatic cancer cells, and breast cancer cells [81,86,87]. Andras et al. found that, except for CD36, the most prevalent FA transporters (such as FABPpm, FATP1, and FATP4) were unaffected by human primary adipocyte coculture [87]. Human primary adipocyte coculture increased CD36 protein levels in tumor cells; furthermore, tumor cells that express high levels of the fatty acid receptor CD36 and lipid metabolism genes are unique in their ability to initiate metastasis [44]. Recent studies have revealed a notable disparity in the expression of CD36 between metastatic and non-metastatic cancer cells, indicating a higher level of CD36 in the former [88]. It seems that CD36 expression affects the metabolism of tumor cells by reducing glucose oxidation and promoting a higher uptake and storage of lipids from the diet, rather than activating the synthesis of in-house lipids. Palmitic acid, or a high-fat diet, explicitly boosts the metastatic potential of CD36^+^ metastasis-initiating cells in a CD36-dependent manner [44]. The metabolic characteristics of normal cells, tumor cells, and metastatic-initiating cells are shown in Figure 2.

## 4. The Functions of CD36

**Figure 3:**
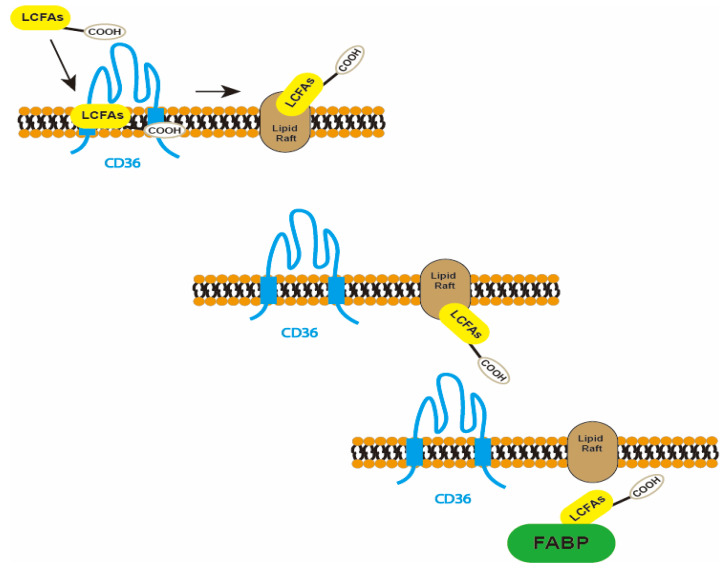
The process of long-chain fatty acids uptake mediated by CD36.

CD36, an extensively glycosylated 80 kDa integral membrane protein, is expressed in a variety of cell types, including platelets, monocyte phagocytes, adipocytes, hepatocytes, myocytes, and certain epithelial cells. CD36 is sometimes called GPIV, GPIIIb, PAS IV, or FAT [43]. CD36 belongs to the scavenger receptor B class 2 (SR-B2) and is a transmembrane glycoprotein with two short intracellular domains, two transmembrane segments, and a large extracellular region [89]. Its intracellular C-terminus can bind to tyrosine kinase and initiate CD36-mediated signaling [43]. CD36 binds and internalizes long-chain fatty acids (LCFAs), and oxidizes low-density lipoprotein (oxLDL), thrombospondin-1, and pathogen-associated molecules. Its extracellular structural domain can bind to CD36-related ligands, such as lipid-associated ligands. LCFAs enter cells and serve as a source of energy, with a carboxyl group and a methyl group present at each end of their molecular structure. As Figure 3 shows, CD36 mediates the uptake of cellular LCFAs in three steps. The first step is extracellular uptake, where CD36 serves as a fatty acid receptor that binds the corresponding LCFAs and connects them to specific lipid raft structures on the plasma membrane. The second step is translocation, where the polar carboxyl groups of fatty acids flip from extracellular to intracellular through the lipid bilayer and relocate. The third step is desorption, where fatty acids enter the cytoplasm from the internal binding site of the plasma membrane and bind specifically to fatty acid-binding proteins (FABPs) [90]. FABPs are a group of intracellular proteins that have the ability to bind and transport fatty acids within cells. These proteins play a crucial role in cellular metabolism by binding to free fatty acids and facilitating their transport to specific organelles or enzymes for further breakdown and utilization. Through their binding and transport functions, FABPs help to regulate the metabolism of fatty acids and ensure their efficient utilization within the cell.

As a central regulator of lipid accumulation, CD36 was first associated with the formation of atherosclerosis (AS). In this chronic inflammatory disease, lipids in the blood are deposited in the intima of arteries, causing the fibrous thickening of the intima, necrosis, and the disintegration of deep tissue in order to form atheromatous material that hardens the arterial wall and narrows the lumen [91]. CD36 is involved in this process by augmenting lipid accumulation and inflammatory vesicle activation [92]. OxLDL, a key molecule [93], binds to CD36 and stimulates macrophages to take themselves up. Then, macrophages transform into foam cells, producing large amounts of inflammatory factors and initiating a series of inflammatory responses, thus driving the development of AS.

Recently, the relationship between CD36 and tumor progression and metastasis has been a concern. CD36 is expressed in tumor cells, microvascular endothelial cells, stromal cells, and immune cells in tumor tissues. However, CD36 levels vary among different cell types [94]. CD36 expression is significantly upregulated in malignant epidermal tumors, such as ovarian cancer, gastric cancer, glioblastoma (GBM), and oral squamous cell carcinoma (OSCC).

### 4.1. High CD36 Expression Promotes Cancer Progression

CD36 is highly expressed in many types of cancer cells and also promotes cancer progression. Pascual reported that a subpopulation of CD44+ cells with a high expression of CD36 and lipid metabolism genes has the ability to initiate metastasis in human OSCC [44]. Another piece of evidence showed that CD36 could promote proliferation and migration in breast cancer cells, pointing out their pro-tumorigenic role in breast cancer [95]. The upregulation of CD36 expression promoted CRC metastasis through the upregulation of MMP28, and increased E-cadherin cleavage [96]. CD36 is highly expressed in GC cells, and its levels positively correlate with migration, invasion, and EMT marker expression in GC cell lines [97]. Of the causes of GC cancer recurrence and metastasis, peritoneal metastasis (PM) is the most common [98]. CD36 expression was upregulated in gastric cancer cells under hypoxic conditions, and the upregulation of CD36 expression promotes the migratory and invasive ability of GC cells and peritoneal tumor growth using exogenous FFA [99]. In vitro experiments showed that CD36 promotes EMT progression in cervical cancer cells by interacting with TGF-β [100]. Omental adipocytes reprogram tumor metabolism by upregulating CD36 in OvCa cells, thereby promoting tumor cell invasion and migration [87]. CD36 plays a protumor role in glioblastoma cancer stem cells [101]. CD36 also assisted in facilitating the proliferation and migration activity of OSCC cells [102]. Luo et al. found that CD36 is highly expressed in HCC and promotes the development of HCC through the Src/PI3K/AKT/mTOR signaling pathway [103]. Another study found that CD36 induced fatty acid uptake through the regulation of AKR1C2, which in turn affected the development of HCC both in vivo and in vitro [104].

### 4.2. CD36 Improves Drug Resistance in Tumors

Tumor drug resistance is a significant cause of the limited effectiveness of tumor treatment and postoperative recovery. Currently, tumor drug resistance can be categorized into intrinsic and acquired resistance, based on the time of emergence [105]. Inherent drug resistance, the natural resistance of tumor cells to a specific antitumor drug, independent of whether they have been exposed to that drug, might result from tumor heterogeneity or the expression of specific mutated oncogenes or tumor suppressor genes in tumor cells that are capable of affecting the expression of drug resistance. Acquired resistance refers to the ability of tumor cells to develop resistance induced by chemotherapeutic agents. Tumor cells might initially be sensitive to a particular chemotherapeutic agent when first encountering it; however, they could develop resistance to the same agent in later treatment, causing tumor relapse. Acquired drug resistance might arise due to the activating of a second proto-oncogene as an emerging driver gene, mutations in the drug target, altered expression levels, or changes in the tumor microenvironment after treatment [105]. Multiple studies identified a role for lipid metabolism in chemotherapy resistance in malignant tumors [106,107,108]. IL-6 has been reported, by Zhang et al. in a study in 2022, to facilitate chemoresistance in acute myelocytic leukemia (AML) through the uptake of FAs, regulated by stat3/CD36FA uptake [109]. After injecting mice with CD36 knockout cells, Zhang et al. also found that the leukemia burden was significantly reduced and, after treating them with cytosine arabinoside (Ara-c), they had a more extended survival time [109]. Interestingly, many patients with pancreatic ductal adenocarcinoma (PDAC) are resistant to gemcitabine; CD36 affects gemcitabine resistance by regulating anti-apoptotic proteins, and high CD36 expression in PDAC is critical for poor prognosis [110]. Overexpression of HER2 is observed in approximately 20% of breast cancers and is associated with more aggressive tumor progression and unfavorable prognosis [111]. Due to the development of drug resistance in tumor cells, current HER2-targeted therapies typically only result in short-term efficacy. CD36 expression is upregulated in lapatinib-resistant cells, facilitating their ability to uptake exogenous fatty acids [112]. In vitro and in vivo experimental studies have demonstrated that inhibiting CD36 can suppress the growth of lapatinib-resistant cells [112].

### 4.3. CD36^+^ Cells Respond to Dietary Lipids and Lead to Metastasis Initiation

Several studies have shown that CD36 is enormously elevated in tumor cells that are cocultured with adipocytes, which means that the expression of CD36 in tumor cells is sensitive to the concentration of fatty acids [44,87]. Furthermore, there was a correlation between increased CD36^+^ cells in oral and metastatic lesions and a high-fat diet in mice. This correlation led to a CD36-dependent mechanism that promoted widespread lymph node metastasis. However, CD36 cells did not generate solitary lymph node metastasis under the same conditions. CD36^+^ cells are not only uniquely capable of initiating metastasis. Still, they can also recapitulate their molecular and cellular heterogeneity from the primary origin, representing bona fide metastasis-initiating cells [44].

However, in contrast to tumor cells, the expression of CD36 is downregulated in tumor microvessels and stroma [94]. For example, high mammographic density (MD) is a significant risk factor associated with an increased incidence of breast cancer, and high MD (but cancer-free) tissue shows reduced levels of CD36 [113]. Therefore, CD36 has different effects on tumor cells and cells in the tumor microenvironment (TME). TME, the site of tumorigenesis and growth, is a complex ecosystem of multiple cellular components, including many endothelial cells, fibroblasts, immune cells, and malignant cells [114]. The functions of immune cells are critical to tumorigenesis. In the next section, our focus will be on examining the impact of CD36 on immune cells within the TME.

## 5. Effects of CD36 on Immune Cells in the TME

### 5.1. CD36 Suppresses T-Cell Activation

The tumor microenvironment is very detrimental to effector T cells, causing them to undergo apoptosis in tumors [115]. However, regulatory T (T_reg_) cells are able to survive and perform their suppressive functions in the TME, suggesting that tumor-infiltrating T_reg_ cells may be able to activate metabolic pathways that help them maintain their functions [116]. In the TME, T_reg_ cells can accumulate at a high frequency, inhibit effector immune cell function, and promote tumor growth [117]. Therefore, targeting T_reg_ cells for anticancer immunotherapy is of great clinical importance. In addition to being crucial to tumor suppression, T_reg_ cells are vital to life because they can maintain immune homeostasis by suppressing immune cell function, thereby preventing spontaneous autoimmunity [118]. Therefore, a high-precision target is needed to destroy T_reg_ cells within the tumor, while preserving T_reg_ cells in nontumor tissues [116]. Wang et al. reported in 2020 that CD36 expression and lipid metabolism could suppress tumor-infiltrating T_reg_ cells on antitumor CD8^+^ T-cell responses [119]. They also pointed out that increased CD36 expression could promote tumor cell survival and accumulation in the tumor microenvironment [119]. Built on the fact that CD36 is only upregulated by T_reg_ cells in tumors, it is safe to infer that CD36 could provide a specific target for the local, rather than systemic, inhibition of T_reg_ cells [119].

The TME is highly immunosuppressive, rendering CD8^+^ T cells dysfunctional [120]. Two studies in 2021 corroborated this statement, showing that CD36 expression could be increased in CD8+ tumor-infiltrating lymphocytes (TILs) in human cancers by the cholesterol that exists in TME, and that such an increase could further increase lipid uptake, accumulation, and peroxidation, leading to CD8^+^ TIL dysfunction [121,122]. Additionally, Ma et al. showed that CD36 expression plays a crucial role in making CD8+ T cells less effective in the anti-tumor process by supplying them with fatty acids, thus leading to a decrease in cytokine production and ferroptosis. Such losses in anti-tumor effects of CD8^+^ T cells could be reduced by inhibiting ferroptosis and using immune checkpoint inhibitors. They also found a high amount of PUFAs in the TME, which reduced the production of CD8^+^ effector T-cell cytokines and enhanced the effect of iron ions. Arachidonic acid (AA, ω-6 polyunsaturated fatty acid) was the most often seen PUFA in tumor tissues, which could induce ferroptosis in CD8^+^ T cells [121]. Notably, AA is frequently present in tissues with albumin or the S100A8/S100A9 complex (neutrophil-derived alarmins), which increases the possibility that this complex may mediate supernatural iron degeneration through CD36 signaling [123]. Xu et al., in their 2021 study, found that oxidized phospholipids could be a crucial TME lipid, triggering CD36-mediated CD8^+^ TIL dysfunction [122]. They found that CD36 promoted oxLDL entry into T cells and induced lipid peroxidation and the downstream activation of p38 kinase. In vitro experiments have proven that effector T-cell function could be restored by inhibiting p38. The restoration of CD8^+^ TIL function has been confirmed by in vivo experiments through the resolution of lipid peroxidation via the overexpression of glutathione peroxidase 4.

In conclusion, T cells in the tumor microenvironment are dysfunctional due to the regulation of CD36, which leads to immune suppression and promotes tumor development.

### 5.2. CD36 Plays an Essential Role in the Polarization of Tumor-Associated Macrophages (TAMs) to the M2 Type

Macrophages are essential natural immune cells that are involved in various physiological and pathological activities of the body. Under normal physiological conditions, antigen-presenting cells (mainly including macrophages and dendritic cells) activate downstream antigen-specific T-cell responses through phagocytosis, antigen presentation, and inflammatory responses, which in turn initiate systemic immune responses and form effective immune memory [124]. Thus, macrophage-mediated immune surveillance plays a crucial role in the early stages of tumor development. However, mounting evidence suggests that tumor-associated macrophages (TAMs) not only promote tumor progression, but also exert negative effects on cancer therapy as the tumor advances [125,126]. Macrophages are highly remodeled and heterogeneous; they are also classified into classically activated M1-type macrophages and, alternatively, activated M2-type macrophages, based on their activation status [127,128]. Under the action of multiple factors in the TME, M1-type and M2-type macrophages can interchange. In the early stage of tumors, macrophages are mainly the M1 type and gradually convert to M2 in order to support tumor growth as the cancer progresses. It is generally accepted that the role of TAMs in cancer is similar to that of M2-type macrophages, acting as tumor promoters [129].

TAMs are the most abundant immune cells in the TME, where they play a crucial role in tumorigenesis and development [130]. In animal tumor models, TAMs are considered to be critical inducers of the angiogenic switch. TAMs are potent sources of VEGF and many other proangiogenic factors, such as semaphorin and S100A family members, titanase-like proteins, bone-bridging proteins, and secreted proteins that are acidic and rich in cysteine [131]. TAMs are closely associated with tumor cell invasion and metastasis. The extracellular matrix (ECM) is a scaffold and barrier for tumor cell migration. TAMs can secrete matrix metalloproteinases (MMPs), serine proteases, and histones in order to mediate ECM degradation and cell–ECM interactions to promote tumor cell invasion and migration. TAMs promote the formation of a microenvironment before metastasis. Evidence shows that the overexpression of CD36 in TAMs increases their ability to take up lipids, which human and mouse tumor tissue macrophages are known to be rich in, and that TAMs can gain energy through FAO, which promotes protumor TAM phenotypes [132]. In addition, CD36 deficiency promotes macrophage polarization into the M1 type. Metabolic reprogramming of FAO upregulation in TAMs depends on the induction of peroxisome proliferator-activated receptor γ (PPAR-γ), and PPAR-γ-dependent FAO mediates M2-like polarization in tumor-associated macrophages. S100A4 is a metastasis-promoting oncoprotein with intense protumor activities, and its expression levels were found to be highly positively correlated with PPAR-γ activation in TAMs. S100A4-PPAR-γ could promote TAMs to increase FA uptake through CD36, thereby upregulating TAM FAO [133]. A study found that CD36 expression is upregulated in metastasis-associated macrophages (MAMs) [134]. MAMs contain a higher abundance of lipid droplets and possess a unique ability to engulf long-chain fatty acids derived from tumor cells, which are transported by extracellular vesicles. Through CD36, the lipid-enriched vesicles are selectively taken up by macrophages, providing a fuel source for their metabolism and initiating tumor-promoting activities [134]. CD36 plays a vital role in the polarization of TAMs to the M2 type.

### 5.3. CD36 Induces Natural Killer (NK) Cell Dysfunction

NK cells have recently received large amounts of attention as an emerging frontier in cancer cell therapy. Due to their antigen-unrestricted tumor cell-killing mechanism, NK cells not only possess the ability to resist tumor escape, resulting from antigenic drift, but also exhibit the potential to deliver broad-spectrum efficacy against heterogeneous tumor types [135]. However, the TME inhibits the antitumor activity of NK cells, and defective NK cell function leads to accelerated tumor formation and growth [136]. For instance, one study concluded that lactate in TME inhibits NK cells by decreasing their cytolytic function [137]. Lactate could also increase the proportion of MDSCs, a group of cells capable of inhibiting natural killer cytotoxicity [137]. After the administration of a high-fat diet to mice, PPAR-α/δ promoted lipid accumulation in NK cells and inhibited mTOR-mediated glycolysis, which is the primary energy pathway for maintaining NK cell function, so that the anti-tumor response of NK cells to melanoma was impaired [138]. It has been demonstrated that NK cell dysfunction following cancer surgery contributes to the promotion of metastasis. [139]. According to a study, it was discovered that CD36 expression in NK cells was upregulated in mice that underwent surgical treatment [140]. This upregulation resulted in enhanced lipid accumulation within NK cells, consequently reducing their ability to effectively lyse tumors in vitro [140]. Human colorectal cancer surgery exhibited similar effects on NK cells compared to mouse cancer surgery. These effects included increased lipid content, elevated CD36 expression, reduced granzyme B and perforin production, and impaired cytotoxicity [140]. Therefore, gaining a comprehensive understanding of the mechanism underlying lipid accumulation in NK cells could significantly improve cancer prognosis.

### 5.4. CD36 Enhances the Immunosuppressive Effect of Myeloid-Derived Suppressor Cells (MDSCs)

MDSCs are potent immunosuppressive cells of bone marrow origin that generally differentiate into macrophages, dendritic cells, and neutrophils [141]. The number of MDSCs in the human body is minimal under normal conditions. However, in pathological states, especially when tumor cells are present in the body, cytokines secreted by tumor cells can promote the production of MDSCs and inhibit the normal differentiation of MDSCs, resulting in the accumulation of MDSCs in the body [142]. MDSCs can suppress T-cell-mediated antitumor immunity and suppress NK cell- and macrophage-mediated innate immunity. MDSCs can also induce the proliferation of T_reg_ cells through various pathways, leaving the tumor microenvironment in an immunosuppressed state [143]. The metabolic shift from glycolysis to lipid metabolism plays a crucial role in regulating the differentiation and function of various subpopulations of bone marrow cells. Recently, it has been documented that tumor-infiltrating MDSCs predominantly rely on FAO as their primary ATP source, thereby sustaining their immunosuppressive capacity [144]. One of the features of this metabolic reprogramming is the increased expression of the lipid uptake receptor CD36 [145]. The metabolic transition from glycolysis to lipid metabolism plays a critical role in modulating the differentiation and function of diverse subsets of bone marrow cells. The genetic deficiency of CD36 genes has been shown to result in the decreased uptake of fatty acids and the reduced content of neutral lipids, leading to a decrease in oxidative metabolism. This deficiency has been observed to contribute to the delayed growth of tumors, which is mediated through CD8^+^ T cells [144].

**Figure 4:**
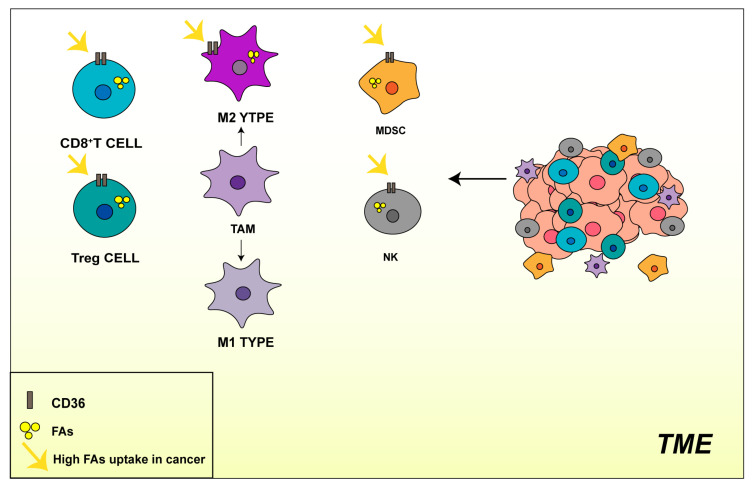
High expression of CD36 by immune cells in the tumor microenvironment promotes the uptake of fatty acids and leads to immunosuppression.

In summary, the reprogramming of fatty acid metabolism is critical in the progression of malignant tumors. On the one hand, the high expression of CD36 in tumor cells triggers fatty acid uptake and lipid accumulation, which helps promote rapid tumor growth and initiate metastasis. On the other hand, immune cells in the TME overexpress CD36 (Figure 4) and undergo metabolic reprogramming. CD36-mediated FA uptake leads to lipid accumulation and immunosuppressive effects. Therefore, preventing CD36 expression or interfering with its function might become a treatment for tumors or, particularly, an effective strategy for inhibiting tumor metastasis.

## 6. CD36 as a Therapeutic Target for Tumors

Currently, many studies have shown that CD36 can be used as a potential target for cancer therapy. There are two reported methods to inhibit the function of CD36 as a lipid transporter: (1) the use of monoclonal antibodies directed to ligand-binding sites; (2) the use of CD36-binding small molecules [146].

Several anti-CD36 antibodies on the market today have been shown to work as CD36 inhibitors to stop tumor progression. Pascual et al. evaluated the role of two CD36 inhibitory antibodies (clone JC63.1 and FA6-152) in an in vivo model of OSCC [44]. They treated NSG mice that were inoculated with OSCC in situ with antibodies, showing that this treatment inhibited tumor metastasis. In addition, their treatment with the CD36 antibody was not toxic to the mice. Furthermore, JC63.1 has demonstrated significant results in treating ovarian cancer, gastric cancer, and bladder cancer [87,147,148]. Clone Ona-0-v1 also exhibited anticancer effects in ovarian, CRC, and oral cancer mouse models [149].

Another approach to modulating CD36 function is using small molecules that could inhibit the activity of the receptor. Several antagonists have been determined to be effective. Nobiletin, a flavonoid extracted from citrus peel, was found to have antitumor effects on CD36-dependent breast cancer cells [150]. In hepatocellular carcinoma cells, sulfo-N-succinimidyl oleate (SSO) reduces the EMT phenotype and decreases the migration rate of PA-treated cells by inhibiting CD36 [151]. SSO reduces the proliferation of primary CRC cells while increasing cleaved caspase-3 levels and reducing xenograft tumor growth [152]. Moreover, 2-methylthio-1,4-naphthoquinone (MTN) is a specific CD36 inhibitor with anticancer activity in GBM [153].

In addition to the methods described above, Jayawardhana et al. designed a Pt(iv) prodrug that mimics the structure of a fatty acid, entering ovarian cancer cells via CD36 and triggering mitochondrial damage, thereby eliminating ovarian cancer cells [154].

## 7. Perspectives

The regulation of CD36 expression by peripheral adipocytes, as well as the specific enhancement of metastatic potential in CD36^+^ metastasis-initiating cells through a high-fat diet, suggests the need to control the quantity and composition of dietary lipids. Concerning daily diet, opting for food choices from the right food categories ensures a balanced and appropriate nutritional intake, thereby offering better alternatives than consuming too little or too much. The prevention of cancer through diet and nutrition is of high practical value. The Mediterranean diet is currently recognized as a healthy diet, characterized by high intakes of vegetables, legumes, fresh fruits, unrefined grains, nuts, and olive oil; moderate intakes of fish and dairy products; low consumption levels of red meat; and moderate consumption levels of alcohol. There is substantial evidence that Mediterranean dietary patterns are negatively associated with cancer risk [155].

In addition to targeting FA uptake, further research is needed to investigate the inhibition of cancer development through other energy source pathways utilized by cancer cells. Additionally, the intervention in tumor development from de novo lipid synthesis pathways requires exploration. Efforts to develop drugs that target tumor metabolism have also gained momentum, with a particular focus on disrupting key metabolic pathways, such as aerobic glycolysis, glutamine metabolism, fatty acid synthesis, nucleotide metabolism, and mitochondrial function. Moreover, preclinical studies of these agents have demonstrated promising results, and several drugs are now being evaluated in clinical trials [156].

**Figure 5:**
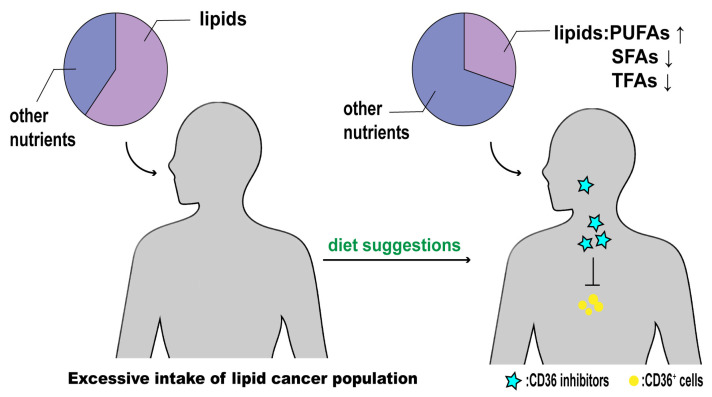
Dietary advice for people who consume too many lipids that cause cancer. Optimize dietary patterns by reducing total lipid intake, increasing polyunsaturated fatty acids, and limiting saturated and trans fatty acids. Simultaneously administer CD36 inhibitors to interfere with the metabolic reprogramming of tumor cells. Implement a diet in conjunction with CD36 inhibitor therapy to inhibit tumor progression and metastasis.

The journey toward effective tumor treatment still has a long way to go. More research targeting CD36 and interfering with FA uptake might be an effective strategy for tumor treatment. In addition to CD36 as a target, controlling dietary lipid intake can be a supplementary therapeutic treatment (Figure 5). Reducing lipid intake and replacing saturated and trans fatty acids with foods rich in polyunsaturated fatty acids can help with tumor treatment. People can incorporate this pattern into their daily diet.

## Figures and Tables

**Table 1:** Cancer-related foods and correlative cancer types. (Cancer types in the table have been ordered according to the correlation between food and cancer risk in the results of the literature).

Food Type	Cancer Type
red meats	Nasopharyngeal cancers [17], endometrial cancer [18], bladder cancer [19], gastric cancer [20], lung cancer [21], NHL [22], breast cancer [23], colorectal cancer [24], and esophageal cancer [25]
processed meats	nasopharyngeal cancer [17], oral and oropharyngeal cancers [26], gastric cancer [20], endometrial cancer [18], bladder cancer [19], NHL [22], breast cancer [27], colorectal cancer [24], esophageal cancer [25], and prostate cancer [28]
sweet beverages	Endometrial cancer [29], liver cncer [30], colorectal cancer and pancreatic cancer [31], thyroid carcinoma [32], prostate cancer [33], and biliary tract cancer [34]
fried foods	prostate cancer [35], gastric cancer [36], lung cancer [37], esophageal cancer [38], oral cancer [39], colorectal cancer [40], and breast cancer [41]
refined grains	gastric cancer [25], breast cancer [20], and colorectal cancer [21]

## Data Availability

No new data were created or analyzed in this study. Data sharing is not applicable to this article.

## References

[1] Cheng C., Geng F., Cheng X., Guo D. (2018). Lipid metabolism reprogramming and its potential targets in cancer. Cancer Commun..

[2] Bechmann L.P., Hannivoort R.A., Gerken G., Hotamisligil G.S., Trauner M., Canbay A. (2012). The interaction of hepatic lipid and glucose metabolism in liver diseases. J. Hepatol..

[3] Butler L.M., Perone Y., Dehairs J., Lupien L.E., de Laat V., Talebi A., Loda M., Kinlaw W.B., Swinnen J.V. (2020). Lipids and cancer: Emerging roles in pathogenesis, diagnosis and therapeutic intervention. Adv. Drug Deliver Rev..

[4] Sun C.Y., Zheng Z.L., Chen C.W., Lu B.W., Liu D. (2022). Targeting Gut Microbiota With Natural Polysaccharides: Effective Interventions Against High-Fat Diet-Induced Metabolic Diseases. Front. Microbiol..

[5] Anderson A.S., Key T.J., Norat T., Scoccianti C., Cecchini M., Berrino F., Boutron-Ruault M.C., Espina C., Leitzmann M., Powers H. (2015). European Code against Cancer 4th Edition: Obesity, body fatness and cancer. Cancer Epidemiol..

[6] Miyamoto J., Igarashi M., Watanabe K., Karaki S.I., Mukouyama H., Kishino S., Li X., Ichimura A., Irie J., Sugimoto Y. (2019). Gut microbiota confers host resistance to obesity by metabolizing dietary polyunsaturated fatty acids. Nat. Commun..

[7] Merritt M.A., Tzoulaki J., van den Brandt P.A., Schouten L.J., Tsilidis K.K., Weiderpass E., Patel C.J., Tjonneland A., Hansen L., Overvad K. (2016). Nutrient-wide association study of 57 foods/nutrients and epithelial ovarian cancer in the European Prospective Investigation into Cancer and Nutrition study and the Netherlands Cohort Study. Am. J. Clin. Nutr..

[8] Wang X., Sun B., Wei L., Jian X., Shan K., He Q., Huang F., Ge X., Gao X., Feng N. (2022). Cholesterol and saturated fatty acids synergistically promote the malignant progression of prostate cancer. Neoplasia.

[9] Ruan L., Cheng S.P., Zhu Q.X. (2020). Dietary Fat Intake and the Risk of Skin Cancer: A Systematic Review and Meta-Analysis of Observational Studies. Nutr. Cancer.

[10] Shetty P.J., Sreedharan J. (2019). Breast Cancer and Dietary Fat Intake: A correlational study. Nepal. J. Epidemiol..

[11] Wirkus J., Ead A.S., Mackenzie G.G. (2021). Impact of dietary fat composition and quantity in pancreatic carcinogenesis: Recent advances and controversies. Nutr. Res..

[12] Zhao L., Deng C., Lin Z., Giovannucci E., Zhang X. (2021). Dietary Fats, Serum Cholesterol and Liver Cancer Risk: A Systematic Review and Meta-Analysis of Prospective Studies. Cancers.

[13] Newman T.M., Vitolins M.Z., Cook K.L. (2019). From the Table to the Tumor: The Role of Mediterranean and Western Dietary Patterns in Shifting Microbial-Mediated Signaling to Impact Breast Cancer Risk. Nutrients.

[14] Lopez-Suarez A. (2019). Burden of cancer attributable to obesity, type 2 diabetes and associated risk factors. Metabolism.

[15] Huang Y., Cao D.H., Chen Z.Y., Chen B., Li J., Guo J.B., Dong Q., Liu L.R., Wei Q. (2021). Red and processed meat consumption and cancer outcomes: Umbrella review. Food Chem..

[16] Chazelas E., Srour B., Desmetz E., Kesse-Guyot E., Julia C., Deschamps V., Druesne-Pecollo N., Galan P., Hercberg S., Latino-Martel P. (2019). Sugary drink consumption and risk of cancer: Results from NutriNet-Sante prospective cohort. BMJ.

[17] Li F., Duan F., Zhao X., Song C., Cui S., Dai L. (2016). Red Meat and Processed Meat Consumption and Nasopharyngeal Carcinoma Risk: A Dose-response Meta-analysis of Observational Studies. Nutr. Cancer.

[18] Bandera E.V., Kushi L.H., Moore D.F., Gifkins D.M., McCullough M.L. (2007). Consumption of animal foods and endometrial cancer risk: A systematic literature review and meta-analysis. Cancer Causes Control..

[19] Crippa A., Larsson S.C., Discacciati A., Wolk A., Orsini N. (2018). Red and processed meat consumption and risk of bladder cancer: A dose-response meta-analysis of epidemiological studies. Eur. J. Nutr..

[20] Kim S.R., Kim K., Lee S.A., Kwon S.O., Lee J.K., Keum N., Park S.M. (2019). Effect of Red, Processed, and White Meat Consumption on the Risk of Gastric Cancer: An Overall and Dose-Response Meta-Analysis. Nutrients.

[21] Yang W.S., Wong M.Y., Vogtmann E., Tang R.Q., Xie L., Yang Y.S., Wu Q.J., Zhang W., Xiang Y.B. (2012). Meat consumption and risk of lung cancer: Evidence from observational studies. Ann. Oncol..

[22] Yang L., Dong J., Jiang S., Shi W., Xu X., Huang H., You X., Liu H. (2015). Red and Processed Meat Consumption Increases Risk for Non-Hodgkin Lymphoma: A PRISMA-Compliant Meta-Analysis of Observational Studies. Medicine.

[23] Guo J., Wei W., Zhan L. (2015). Red and processed meat intake and risk of breast cancer: A meta-analysis of prospective studies. Breast Cancer Res. Treat..

[24] Schwingshackl L., Schwedhelm C., Hoffmann G., Knuppel S., Laure Preterre A., Iqbal K., Bechthold A., De Henauw S., Michels N., Devleesschauwer B. (2018). Food groups and risk of colorectal cancer. Int. J. Cancer.

[25] Zhu H.C., Yang X., Xu L.P., Zhao L.J., Tao G.Z., Zhang C., Qin Q., Cai J., Ma J.X., Mao W.D. (2014). Meat Consumption Is Associated with Esophageal Cancer Risk in a Meat- and Cancer-Histological-Type Dependent Manner. Digest Dis. Sci..

[26] Xu J., Yang X.X., Wu Y.G., Li X.Y., Bai B. (2014). Meat consumption and risk of oral cavity and oropharynx cancer: A meta-analysis of observational studies. PLoS ONE.

[27] Farvid M.S., Stern M.C., Norat T., Sasazuki S., Vineis P., Weijenberg M.P., Wolk A., Wu K., Stewart B.W., Cho E. (2018). Consumption of red and processed meat and breast cancer incidence: A systematic review and meta-analysis of prospective studies. Int. J. Cancer.

[28] Bylsma L.C., Alexander D.D. (2015). A review and meta-analysis of prospective studies of red and processed meat, meat cooking methods, heme iron, heterocyclic amines and prostate cancer. Nutr. J..

[29] Inoue-Choi M., Robien K., Mariani A., Cerhan J.R., Anderson K.E. (2013). Sugar-Sweetened Beverage Intake and the Risk of Type I and Type II Endometrial Cancer among Postmenopausal Women. Cancer Epidemiol. Biomarkers Prev..

[30] Stepien M., Duarte-Salles T., Fedirko V., Trichopoulou A., Lagiou P., Bamia C., Overvad K., Tjonneland A., Hansen L., Boutron-Ruault M.C. (2016). Consumption of soft drinks and juices and risk of liver and biliary tract cancers in a European cohort. Eur. J. Nutr..

[31] Llaha F., Gil-Lespinard M., Unal P., de Villasante I., Castaneda J., Zamora-Ros R. (2021). Consumption of Sweet Beverages and Cancer Risk. A Systematic Review and Meta-Analysis of Observational Studies. Nutrients.

[32] Zamora-Ros R., Beraud V., Franceschi S., Cayssials V., Tsilidis K.K., Boutron-Ruault M.C., Weiderpass E., Overvad K., Tjonneland A., Eriksen A.K. (2018). Consumption of fruits, vegetables and fruit juices and differentiated thyroid carcinoma risk in the European Prospective Investigation into Cancer and Nutrition (EPIC) study. Int. J. Cancer.

[33] Miles F.L., Neuhouser M.L., Zhang Z.F. (2018). Concentrated sugars and incidence of prostate cancer in a prospective cohort. Br. J. Nutr..

[34] Larsson S.C., Giovannucci E.L., Wolk A. (2016). Sweetened Beverage Consumption and Risk of Biliary Tract and Gallbladder Cancer in a Prospective Study. J. Natl. Cancer Inst..

[35] Lippi G., Mattiuzzi C. (2015). Fried food and prostate cancer risk: Systematic review and meta-analysis. Int. J. Food Sci. Nutr..

[36] Guo L.W., Liu S.Z., Zhang M., Chen Q., Zhang S.K., Sun X.B. (2018). Multivariate analysis of the association between consumption of fried food and gastric cancer and precancerous lesions. Zhōnghuá Yùfáng-Yīxué Zázhì.

[37] Yu H., Xu Q., Xiong W., Liu Z., Cai L., He F. (2019). Association of pickled food, fired food and smoked food combined with smoking and alcohol drinking with lung cancer: A case-control study. Wei Sheng Yan Jiu.

[38] Guo L., Liu S., Zhang M., Chen Q., Zhang S., Sun X. (2017). Multivariate ordinal logistic regression analysis on the association between consumption of fried food and both esophageal cancer and precancerous lesions. Zhong Guo Di Fang Bing Xue Za Ji.

[39] Rodriguez-Molinero J., Miguelanez-Medran B.D., Puente-Gutierrez C., Delgado-Somolinos E., Carreras-Presas C.M., Fernandez-Farhall J., Lopez-Sanchez A.F. (2021). Association between Oral Cancer and Diet: An Update. Nutrients.

[40] Saadati H.M., Okhovat B., Khodamoradi F. (2021). Incidence and Risk Factors of Colorectal Cancer in the Iranian Population: A Systematic Review. J. Gastrointest. Cancer.

[41] Ganesan K., Xu B.J. (2020). Deep frying cooking oils promote the high risk of metastases in the breast-A critical review. Food Chem. Toxicol..

[42] Das U.N. (2019). Saturated Fatty Acids, MUFAs and PUFAs Regulate Ferroptosis. Cell Chem. Biol..

[43] Pepino M.Y., Kuda O., Samovski D., Abumrad N.A. (2014). Structure-Function of CD36 and Importance of Fatty Acid Signal Transduction in Fat Metabolism. Annu. Rev. Nutr..

[44] Pascual G., Avgustinova A., Mejetta S., Martin M., Castellanos A., Attolini C.S.O., Berenguer A., Prats N., Toll A., Hueto J.A. (2017). Targeting metastasis-initiating cells through the fatty acid receptor CD36. Nature.

[45] Fan Y., Qiu Y., Wang J., Chen Q., Wang S.J., Wang Y.P., Li Y.N., Weng Y.F., Qian J.W., Chen F. (2022). Association Between Dietary Fatty Acid Pattern and Risk of Oral Cancer. Front. Nutr..

[46] Yang J.J., Yu D., Takata Y., Smith-Warner S.A., Blot W., White E., Robien K., Park Y., Xiang Y.B., Sinha R. (2017). Dietary Fat Intake and Lung Cancer Risk: A Pooled Analysis. J. Clin. Oncol..

[47] Xia H., Ma S., Wang S., Sun G. (2015). Meta-Analysis of Saturated Fatty Acid Intake and Breast Cancer Risk. Medicine.

[48] Binker-Cosen M.J., Richards D., Oliver B., Gaisano H.Y., Binker M.G., Cosen-Binker L.I. (2017). Palmitic acid increases invasiveness of pancreatic cancer cells AsPC-1 through TLR4/ROS/NF-κB/MMP-9 signaling pathway. Biochem. Biophys. Res. Commun..

[49] Bojkova B., Winklewski P.J., Wszedybyl-Winklewska M. (2020). Dietary Fat and Cancer-Which Is Good, Which Is Bad, and the Body of Evidence. Int. J. Mol. Sci..

[50] Pan J., Fan Z., Wang Z., Dai Q., Xiang Z., Yuan F., Yan M., Zhu Z., Liu B., Li C. (2019). CD36 mediates palmitate acid-induced metastasis of gastric cancer via AKT/GSK-3beta/beta-catenin pathway. J. Exp. Clin. Cancer Res..

[51] Fatima S., Hu X., Huang C., Zhang W., Cai J., Huang M., Gong R.H., Chen M., Ho A.H.M., Su T. (2019). High-fat diet feeding and palmitic acid increase CRC growth in beta2AR-dependent manner. Cell Death Dis..

[52] Yang P., Su C., Luo X., Zeng H., Zhao L., Wei L., Zhang X., Varghese Z., Moorhead J.F., Chen Y. (2018). Dietary oleic acid-induced CD36 promotes cervical cancer cell growth and metastasis via up-regulation Src/ERK pathway. Cancer Lett..

[53] Gaston R., Maria Eugenia P., Das U.N., Eynard A.R. (2017). Polyunsaturated Fatty Acids Differentially Modulate Cell Proliferation and Endocannabinoid System in Two Human Cancer Lines. Arch. Med. Res..

[54] Xiang F., Wu K., Liu Y., Shi L., Wang D., Li G., Tao K., Wang G. (2017). Omental adipocytes enhance the invasiveness of gastric cancer cells by oleic acid-induced activation of the PI3K-Akt signaling pathway. Int. J. Biochem. Cell Biol..

[55] Liotti A., Cosimato V., Mirra P., Cali G., Conza D., Secondo A., Luongo G., Terracciano D., Formisano P., Beguinot F. (2018). Oleic acid promotes prostate cancer malignant phenotype via the G protein-coupled receptor FFA1/GPR40. J. Cell Physiol..

[56] Sanchez-Quesada C., Lopez-Biedma A., Warleta F., Campos M., Beltran G., Gaforio J.J. (2013). Bioactive Properties of the Main Triterpenes Found in Olives, Virgin Olive Oil, and Leaves of Olea europaea. J. Agric. Food Chem..

[57] Saini R.K., Keum Y.S. (2018). Omega-3 and omega-6 polyunsaturated fatty acids: Dietary sources, metabolism, and significance—A review. Life Sci..

[58] Glaser C., Heinrich J., Koletzko B. (2010). Role of FADS1 and FADS2 polymorphisms in polyunsaturated fatty acid metabolism. Metabolism.

[59] Cheon E.C., Strouch M.J., Barron M.R., Ding Y., Melstrom L.G., Krantz S.B., Mullapudi B., Adrian K., Rao S., Adrian T.E. (2011). Alteration of strain background and a high omega-6 fat diet induces earlier onset of pancreatic neoplasia in EL-Kras transgenic mice. Int. J. Cancer.

[60] Kawahara I., Mori T., Goto K., Fujii K., Ohmori H., Kishi S., Fujiwara-Tani R., Kuniyasu H. (2017). Fatty Acids Induce Stemness in the Stromal Cells of a CT26 Mouse Tumor Model. Pathobiology.

[61] Matsuoka T., Adair J.E., Lih F.B., Hsi L.C., Rubino M., Eling T.E., Tomer K.B., Yashiro M., Hirakawa K., Olden K. (2010). Elevated dietary linoleic acid increases gastric carcinoma cell invasion and metastasis in mice. Br. J. Cancer.

[62] Serna-Marquez N., Diaz-Aragon R., Reyes-Uribe E., Cortes-Reynosa P., Salazar E.P. (2017). Linoleic acid induces migration and invasion through FFAR4-and PI3K-/Akt-dependent pathway in MDA-MB-231 breast cancer cells. Med. Oncol..

[63] Espinosa-Neira R., Mejia-Rangel J., Cortes-Reynosa P., Salazar E.P. (2011). Linoleic acid induces an EMT-like process in mammary epithelial cells MCF10A. Int. J. Biochem. Cell B.

[64] Moussa I., Day R.S., Li R.S., Kaseb A., Jalal P.K., Daniel-MacDougall C., Hatia R.I., Abdelhakeem A., Rashid A., Chun Y.S. (2021). Association of dietary fat intake and hepatocellular carcinoma among US adults. Cancer Med.-US.

[65] Ma J.C., Zhang C.S., Liang W.Q., Li L., Du J., Pan C.W., Chen B.L., Chen Y.Z., Wang Y.P. (2022). omega-3 and omega-6 Polyunsaturated Fatty Acids Regulate the Proliferation, Invasion and Angiogenesis of Gastric Cancer Through COX/PGE Signaling Pathway. Front. Oncol..

[66] Dimri M., Bommi P.V., Sahasrabuddhe A.A., Khandekar J.D., Dimri G.P. (2010). Dietary omega-3 polyunsaturated fatty acids suppress expression of EZH2 in breast cancer cells. Carcinogenesis.

[67] Lee H.C., Liang A., Lin Y.H., Guo Y.R., Huang S.Y. (2018). Low dietary n-6/n-3 polyunsaturated fatty acid ratio prevents induced oral carcinoma in a hamster pouch model. Prostag. Leukotr. Ess..

[68] Nindrea R.D., Aryandono T., Lazuardi L., Dwiprahasto I. (2019). Association of Dietary Intake Ratio of n-3/n-6 Polyunsaturated Fatty Acids with Breast Cancer Risk in Western and Asian Countries: A Meta-Analysis. Asian Pac. J. Cancer Prev..

[69] Michels N., Specht I.O., Heitmann B.L., Chajes V., Huybrechts I. (2021). Dietary trans-fatty acid intake in relation to cancer risk: A systematic review and meta-analysis. Nutr. Rev..

[70] Islam M.A., Amin M.N., Siddiqui S.A., Hossain M.P., Sultana F., Kabir M.R. (2019). Trans fatty acids and lipid profile: A serious risk factor to cardiovascular disease, cancer and diabetes. Diabetes Metab. Synd..

[71] Matta M., Huybrechts I., Biessy C., Casagrande C., Yammine S., Fournier A., Olsen K.S., Lukic M., Gram I.T., Ardanaz E. (2021). Dietary intake of trans fatty acids and breast cancer risk in 9 European countries. BMC Med..

[72] Yammine S., Huybrechts I., Biessy C., Dossus L., Aglago E.K., Naudin S., Ferrari P., Weiderpass E., Tjonneland A., Hansen L. (2020). Dietary and Circulating Fatty Acids and Ovarian Cancer Risk in the European Prospective Investigation into Cancer and Nutrition. Cancer Epidemiol. Biomark. Prev..

[73] Fujii K., Luo Y., Fujiwara-Tani R., Kishi S., He S., Yang S.Y., Sasaki T., Ohmori H., Kuniyasu H. (2017). Pro-metastatic intracellular signaling of the elaidic trans fatty acid. Int. J. Oncol..

[74] Korat A.V.A., Chiu Y.H., Bertrand K.A., Zhang S.M., Epstein M.M., Rosner B.A., Chiuve S., Campos H., Giovannucci E.L., Chavarro J.E. (2020). Red blood cell membrane trans fatty acid levels and risk of non-Hodgkin lymphoma: A prospective nested case-control study. Am. J. Clin. Nutr..

[75] Hu X., Wang X.J., Jia F.P., Tanaka N., Kimura T., Nakajima T., Sato Y., Moriya K., Koike K., Gonzalez F.J. (2020). A trans-fatty acid-rich diet promotes liver tumorigenesis in HCV core gene transgenic mice. Carcinogenesis.

[76] Guerra L., Bonetti L., Brenner D. (2020). Metabolic Modulation of Immunity: A New Concept in Cancer Immunotherapy. Cell Rep..

[77] Corn K.C., Windham M.A., Rafat M. (2020). Lipids in the tumor microenvironment: From cancer progression to treatment. Prog. Lipid Res..

[78] Vander Heiden M.G., DeBerardinis R.J. (2017). Understanding the Intersections between Metabolism and Cancer Biology. Cell.

[79] Broadfield L.A., Pane A.A., Talebi A., Swinnen J.V., Fendt S.M. (2021). Lipid metabolism in cancer: New perspectives and emerging mechanisms. Dev. Cell.

[80] Brown M., Assen F.P., Leithner A., Abe J., Schachner H., Asfour G., Bago-Horvath Z., Stein J.V., Uhrin P., Sixt M. (2018). Lymph node blood vessels provide exit routes for metastatic tumor cell dissemination in mice. Science.

[81] Haidari S., Troltzsch M., Knosel T., Liokatis P., Kasintsova A., Eberl M., Ortner F., Otto S., Fegg F., Boskov M. (2021). Fatty Acid Receptor CD36 Functions as a Surrogate Parameter for Lymph Node Metastasis in Oral Squamous Cell Carcinoma. Cancers.

[82] Lee C.K., Jeong S.H., Jang C., Bae H., Kim Y.H., Park I., Kim S.K., Koh G.Y. (2019). Tumor metastasis to lymph nodes requires YAP-dependent metabolic adaptation. Science.

[83] Ohshima K., Morii E. (2021). Metabolic Reprogramming of Cancer Cells during Tumor Progression and Metastasis. Metabolites.

[84] Koundouros N., Poulogiannis G. (2020). Reprogramming of fatty acid metabolism in cancer. Brit. J. Cancer.

[85] Ruan C.W., Meng Y.K., Song H. (2022). CD36: An emerging therapeutic target for cancer and its molecular mechanisms. J. Cancer Res. Clin..

[86] Wright H.J., Hou J., Xu B., Cortez M., Potma E.O., Tromberg B.J., Razorenova O.V. (2017). CDCP1 drives triple-negative breast cancer metastasis through reduction of lipid-droplet abundance and stimulation of fatty acid oxidation. Proc. Natl. Acad. Sci. USA.

[87] Ladanyi A., Mukherjee A., Kenny H.A., Johnson A., Mitra A.K., Sundaresan S., Nieman K.M., Pascual G., Benitah S.A., Montag A. (2018). Adipocyte-induced CD36 expression drives ovarian cancer progression and metastasis. Oncogene.

[88] Bergers G., Fendt S.M. (2021). The metabolism of cancer cells during metastasis. Nat. Rev. Cancer.

[89] Armesilla A.L., Vega M.A. (1994). Structural organization of the gene for human CD36 glycoprotein. J. Biol. Chem..

[90] Luo D., Wang S., Zhao X., Han X.F. (2019). Fatty Acid Translocase CD36/SR-B2 and Its Mediation in Transmembrane Transportation of Long-Chain Fatty Acids. Chin. J. Anim. Nutr..

[91] Zhu Y.H., Xian X.M., Wang Z.Z., Bi Y.C., Chen Q.G., Han X.F., Tang D.Q., Chen R.J. (2018). Research Progress on the Relationship between Atherosclerosis and Inflammation. Biomolecules.

[92] Wang Y.T., Fang C.Y., Xu L., Yang B.W., Song E.Q., Song Y. (2021). Polybrominated Diphenyl Ether Quinone Exposure Induces Atherosclerosis Progression via CD36-Mediated Lipid Accumulation, NLRP3 Inflammasome Activation, and Pyroptosis. Chem. Res. Toxicol..

[93] Zingg J.M., Vlad A., Ricciarelli R. (2021). Oxidized LDLs as Signaling Molecules. Antioxidants.

[94] Wang J.C., Li Y.S. (2019). CD36 tango in cancer: Signaling pathways and functions. Theranostics.

[95] Liang Y., Han H., Liu L., Duan Y., Yang X., Ma C., Zhu Y., Han J., Li X., Chen Y. (2018). CD36 plays a critical role in proliferation, migration and tamoxifen-inhibited growth of ER-positive breast cancer cells. Oncogenesis.

[96] Drury J., Rychahou P.G., Kelson C.O., Geisen M.E., Wu Y., He D., Wang C., Lee E.Y., Evers B.M., Zaytseva Y.Y. (2022). Upregulation of CD36, a Fatty Acid Translocase, Promotes Colorectal Cancer Metastasis by Increasing MMP28 and Decreasing E-Cadherin Expression. Cancers.

[97] Wang J., Wen T., Li Z., Che X., Gong L., Jiao Z., Qu X., Liu Y. (2020). CD36 upregulates DEK transcription and promotes cell migration and invasion via GSK-3beta/beta-catenin-mediated epithelial-to-mesenchymal transition in gastric cancer. Aging.

[98] Thomassen I., van Gestel Y.R., van Ramshorst B., Luyer M.D., Bosscha K., Nienhuijs S.W., Lemmens V.E., de Hingh I.H. (2014). Peritoneal carcinomatosis of gastric origin: A population-based study on incidence, survival and risk factors. Int. J. Cancer.

[99] Aoki T., Kinoshita J., Munesue S., Hamabe-Horiike T., Yamaguchi T., Nakamura Y., Okamoto K., Moriyama H., Nakamura K., Harada S. (2023). Hypoxia-Induced CD36 Expression in Gastric Cancer Cells Promotes Peritoneal Metastasis via Fatty Acid Uptake. Ann. Surg. Oncol..

[100] Deng M., Cai X., Long L., Xie L., Ma H., Zhou Y., Liu S., Zeng C. (2019). CD36 promotes the epithelial-mesenchymal transition and metastasis in cervical cancer by interacting with TGF-beta. J. Transl. Med..

[101] Hale J.S., Otvos B., Sinyuk M., Alvarado A.G., Hitomi M., Stoltz K., Wu Q., Flavahan W., Levison B., Johansen M.L. (2014). Cancer stem cell-specific scavenger receptor CD36 drives glioblastoma progression. Stem Cells.

[102] Sakurai K., Tomihara K., Yamazaki M., Heshiki W., Moniruzzaman R., Sekido K., Tachinami H., Ikeda A., Imaue S., Fujiwara K. (2020). CD36 expression on oral squamous cell carcinoma cells correlates with enhanced proliferation and migratory activity. Oral Dis..

[103] Luo X.Q., Zheng E.Z., Wei L., Zeng H., Qin H., Zhang X.Y., Liao M., Chen L., Zhao L., Ruan X.Z. (2021). The fatty acid receptor CD36 promotes HCC progression through activating Src/PI3K/AKT axis-dependent aerobic glycolysis. Cell Death Dis..

[104] Tao L.D., Ding X.M., Yan L.L., Xu G.C., Zhang P.J., Ji A.L., Zhang L.H. (2022). CD36 accelerates the progression of hepatocellular carcinoma by promoting FAs absorption. Med. Oncol..

[105] Wang X., Zhang H.Y., Chen X.Z. (2019). Drug resistance and combating drug resistance in cancer. Cancer Drug Resist..

[106] Lin D., Zhang H.Y., Liu R., Deng T., Ning T., Bai M., Yang Y.C., Zhu K.G., Wang J.Y., Duan J.J. (2021). iRGD-modified exosomes effectively deliver CPT1A siRNA to colon cancer cells, reversing oxaliplatin resistance by regulating fatty acid oxidation. Mol. Oncol..

[107] He W.M., Liang B.S., Wang C.L., Li S.W., Zhao Y., Huang Q., Liu Z.X., Yao Z.Q., Wu Q.J., Liao W.J. (2019). MSC-regulated lncRNA MACC1-AS1 promotes stemness and chemoresistance through fatty acid oxidation in gastric cancer. Oncogene.

[108] Tabe Y., Konopleva M., Andreeff M. (2020). Fatty Acid Metabolism, Bone Marrow Adipocytes, and AML. Front. Oncol..

[109] Zhang Y.J., Guo H.Z., Zhang Z.L., Lu W., Zhu J., Shi J. (2022). IL-6 promotes chemoresistance via upregulating CD36 mediated fatty acids uptake in acute myeloid leukemia. Exp. Cell Res..

[110] Kubo M., Gotoh K., Eguchi H., Kobayashi S., Iwagami Y., Tomimaru Y., Akita H., Asaoka T., Noda T., Takeda Y. (2020). Impact of CD36 on Chemoresistance in Pancreatic Ductal Adenocarcinoma. Ann. Surg. Oncol..

[111] Kumar-Sinha C., Ignatoski K.W., Lippman M.E., Ethier S.P., Chinnaiyan A.M. (2003). Transcriptome analysis of HER2 reveals a molecular connection to fatty acid synthesis. Cancer Res..

[112] Feng W.W., Wilkins O., Bang S., Ung M., Li J., An J., Del Genio C., Canfield K., DiRenzo J., Wells W. (2019). CD36-Mediated Metabolic Rewiring of Breast Cancer Cells Promotes Resistance to HER2-Targeted Therapies. Cell Rep..

[113] DeFilippis R.A., Chang H., Dumont N., Rabban J.T., Chen Y.Y., Fontenay G.V., Berman H.K., Gauthier M.L., Zhao J., Hu D. (2012). CD36 repression activates a multicellular stromal program shared by high mammographic density and tumor tissues. Cancer Discov..

[114] Ma J., Huang L., Hu D., Zeng S., Han Y., Shen H. (2021). The role of the tumor microbe microenvironment in the tumor immune microenvironment: Bystander, activator, or inhibitor?. J. Exp. Clin. Cancer Res..

[115] Horton B.L., Williams J.B., Cabanov A., Spranger S., Gajewski T.F. (2018). Intratumoral CD8(+) T-cell Apoptosis Is a Major Component of T-cell Dysfunction and Impedes Antitumor Immunity. Cancer Immunol. Res..

[116] Horton B.L., Spranger S. (2020). CD36—The Achilles’ heel of Treg cells. Nat. Immunol..

[117] Bos P.D., Plitas G., Rudra D., Lee S.Y., Rudensky A.Y. (2013). Transient regulatory T cell ablation deters oncogene-driven breast cancer and enhances radiotherapy. J. Exp. Med..

[118] Kim J.M., Rasmussen J.P., Rudensky A.Y. (2007). Regulatory T cells prevent catastrophic autoimmunity throughout the lifespan of mice. Nat. Immunol..

[119] Wang H., Franco F., Tsui Y.C., Xie X., Trefny M.P., Zappasodi R., Mohmood S.R., Fernandez-Garcia J., Tsai C.H., Schulze I. (2020). CD36-mediated metabolic adaptation supports regulatory T cell survival and function in tumors. Nat. Immunol..

[120] Mohamed E., Al-Khami A.A., Rodriguez P.C. (2018). The cellular metabolic landscape in the tumor milieu regulates the activity of myeloid infiltrates. Cell Mol. Immunol..

[121] Ma X.Z., Xiao L.L., Liu L.T., Ye L.Q., Su P., Bi E.G., Wang Q., Yang M.J., Qian J.F., Yi Q. (2021). CD36-mediated ferroptosis dampens intratumoral CD8(+) T cell effector function and impairs their antitumor ability. Cell Metab..

[122] Xu S., Chaudhary O., Rodriguez-Morales P., Sun X., Chen D., Zappasodi R., Xu Z., Pinto A.F.M., Williams A., Schulze I. (2021). Uptake of oxidized lipids by the scavenger receptor CD36 promotes lipid peroxidation and dysfunction in CD8(+) T cells in tumors. Immunity.

[123] Subramanian M., Marelli-Berg F.M. (2021). CD36 pumps fat to defang killer T cells in tumors. Cell Metab..

[124] Jhunjhunwala S., Hammer C., Delamarre L. (2021). Antigen presentation in cancer: Insights into tumour immunogenicity and immune evasion. Nat. Rev. Cancer.

[125] Christofides A., Strauss L., Yeo A., Cao C., Charest A., Boussiotis V.A. (2022). The complex role of tumor-infiltrating macrophages. Nat. Immunol..

[126] Binnewies M., Roberts E.W., Kersten K., Chan V., Fearon D.F., Merad M., Coussens L.M., Gabrilovich D.I., Ostrand-Rosenberg S., Hedrick C.C. (2018). Understanding the tumor immune microenvironment (TIME) for effective therapy. Nat. Med..

[127] Mantovani A., Sica A. (2010). Macrophages, innate immunity and cancer: Balance, tolerance, and diversity. Curr. Opin. Immunol..

[128] Muraille E., Leo O., Moser M. (2014). Th1/Th2 paradigm extended: Macrophage polarization as an unappreciated pathogen-driven escape mechanism?. Front. Immunol..

[129] (2022). Tumor Associated Macrophages Protect Colon Cancer Cells from TRAIL-Induced Apoptosis through IL-1 beta-Dependent Stabilization of Snail in Tumor Cells (Expression of Concern of Vol 5, art no E11700, 2010). PLoS ONE.

[130] Sawa-Wejksza K., Kandefer-Szerszen M. (2018). Tumor-Associated Macrophages as Target for Antitumor Therapy. Arch. Immunol. Ther. Ex..

[131] Larionova I., Kazakova E., Gerashchenko T., Kzhyshkowska J. (2021). New Angiogenic Regulators Produced by TAMs: Perspective for Targeting Tumor Angiogenesis. Cancers.

[132] Su P., Wang Q., Bi E.G., Ma X.Z., Liu L.T., Yang M.J., Qian J.F., Yi Q. (2020). Enhanced Lipid Accumulation and Metabolism Are Required for the Differentiation and Activation of Tumor-Associated Macrophages. Cancer Res..

[133] Liu S.Q., Zhang H.L., Li Y.A., Zhang Y.N., Bian Y.Y., Zeng Y.Q., Yao X.H., Wan J.J., Chen X., Li J.R. (2021). S100A4 enhances protumor macrophage polarization by control of PPAR-gamma-dependent induction of fatty acid oxidation. J. Immunother. Cancer.

[134] Yang P., Qin H., Li Y.Y., Xiao A.H., Zheng E.Z., Zeng H., Su C.X., Luo X.Q., Lu Q.N., Liao M. (2022). CD36-mediated metabolic crosstalk between tumor cells and macrophages affects liver metastasis. Nat. Commun..

[135] Cerwenka A., Lanier L.L. (2018). Natural killers join the fight against cancer An antibody overcomes cancer cell immune evasion and activates natural killer cells. Science.

[136] Liu Y., Cheng Y., Xu Y., Wang Z., Du X., Li C., Peng J., Gao L., Liang X., Ma C. (2017). Increased expression of programmed cell death protein 1 on NK cells inhibits NK-cell-mediated anti-tumor function and indicates poor prognosis in digestive cancers. Oncogene.

[137] Certo M., Tsai C.H., Pucino V., Ho P.C., Mauro C. (2021). Lactate modulation of immune responses in inflammatory versus tumour microenvironments. Nat. Rev. Immunol..

[138] Michelet X., Dyck L., Hogan A., Loftus R.M., Duquette D., Wei K., Beyaz S., Tavakkoli A., Foley C., Donnelly R. (2018). Metabolic reprogramming of natural killer cells in obesity limits antitumor responses. Nat. Immunol..

[139] Tai L.H., de Souza C.T., Belanger S., Ly L., Alkayyal A.A., Zhang J., Rintoul J.L., Ananth A.A., Lam T., Breitbach C.J. (2013). Preventing postoperative metastatic disease by inhibiting surgery-induced dysfunction in natural killer cells. Cancer Res..

[140] Niavarani S.R., Lawson C., Bakos O., Boudaud M., Batenchuk C., Rouleau S., Tai L.H. (2019). Lipid accumulation impairs natural killer cell cytotoxicity and tumor control in the postoperative period. BMC Cancer.

[141] Dorhoi A., Du Plessis N. (2018). Monocytic Myeloid-Derived Suppressor Cells in Chronic Infections. Front. Immunol..

[142] Pawelec G., Verschoor C.P., Ostrand-Rosenberg S. (2019). Myeloid-Derived Suppressor Cells: Not Only in Tumor Immunity. Front. Immunol..

[143] Barnie P.A., Zhang P., Lv H.X., Wang D., Su X.L., Su Z.L., Xu H.A. (2017). Myeloid-derived suppressor cells and myeloid regulatory cells in cancer and autoimmune disorders. Exp. Ther. Med..

[144] Al-Khami A.A., Zheng L.Q., Del Valle L., Hossain F., Wyczechowska D., Zabaleta J., Sanchez M.D., Dean M.J., Rodriguez P.C., Ochoa A.C. (2017). Exogenous lipid uptake induces metabolic and functional reprogramming of tumor-associated myeloid-derived suppressor cells. Oncoimmunology.

[145] Al-Khami A.A., Rodriguez P.C., Ochoa A.C. (2016). Metabolic reprogramming of myeloid-derived suppressor cells (MDSC) in cancer. Oncoimmunology.

[146] Guerrero-Rodriguez S.L., Mata-Cruz C., Perez-Tapia S.M., Velasco-Velazquez M.A. (2022). Role of CD36 in cancer progression, stemness, and targeting. Front. Cell Dev. Biol..

[147] Yang L., Sun J.Y., Li M.Q., Long Y.M., Zhang D.Z., Guo H.Q., Huang R.M., Yan J. (2021). Oxidized Low-Density Lipoprotein Links Hypercholesterolemia and Bladder Cancer Aggressiveness by Promoting Cancer Stemness. Cancer Res..

[148] Jiang M.Z., Wu N., Xu B., Chu Y., Li X.W., Su S., Chen D., Li W.J., Shi Y.T., Gao X.L. (2019). Fatty acid-induced CD36 expression via O-GlcNAcylation drives gastric cancer metastasis. Theranostics.

[149] Mokhtari R.B., Homayouni T.S., Baluch N., Morgatskaya E., Kumar S., Das B., Yeger H. (2017). Combination therapy in combating cancer. Oncotarget.

[150] Sp N., Kang D.Y., Kim D.H., Park J.H., Lee H.G., Kim H.J., Darvin P., Park Y.-M., Yang Y.M. (2018). Nobiletin inhibits CD36-dependent tumor angiogenesis, migration, invasion, and sphere formation through the Cd36/Stat3/Nf-Kb signaling axis. Nutrients.

[151] Nath A., Li I., Roberts L.R., Chan C. (2015). Elevated free fatty acid uptake via CD36 promotes epithelial-mesenchymal transition in hepatocellular carcinoma. Sci. Rep..

[152] Drury J., Rychahou P.G., He D., Jafari N., Wang C., Lee E.Y., Weiss H.L., Evers B.M., Zaytseva Y.Y. (2020). Inhibition of Fatty Acid Synthase Upregulates Expression of CD36 to Sustain Proliferation of Colorectal Cancer Cells. Front. Oncol..

[153] Muller W.E., Thakur N.L., Ushijima H., Thakur A.N., Krasko A., Le Pennec G., Indap M.M., Perovic-Ottstadt S., Schroder H.C., Lang G. (2004). Matrix-mediated canal formation in primmorphs from the sponge Suberites domuncula involves the expression of a CD36 receptor-ligand system. J. Cell Sci..

[154] Jayawardhana A.M.D.S., Stilgenbauer M., Datta P., Qiu Z.H., Mckenzie S., Wang H., Bowers D., Kurokawa M., Zheng Y.R. (2020). Fatty acid-like Pt(iv) prodrugs overcome cisplatin resistance in ovarian cancer by harnessing CD36. Chem. Commun..

[155] Almanza-Aguilera E., Cano A., Gil-Lespinard M., Burguera N., Zamora-Ros R., Agudo A., Farràs M. (2023). Mediterranean diet and olive oil, microbiota, and obesity-related cancers. From mechanisms to prevention. Semin. Cancer Biol..

[156] Stine Z.E., Schug Z.T., Salvino J.M., Dang C.V. (2022). Targeting cancer metabolism in the era of precision oncology. Nat. Rev. Drug Discov..

